# Novel Alzheimer’s disease blood‐based biomarkers in exercise trials

**DOI:** 10.1002/alz.088042

**Published:** 2025-01-09

**Authors:** Thomas K Karikari, Kirk I. Erickson, Irene Esteban‐Cornejo

**Affiliations:** ^1^ Department of Psychiatry, School of Medicine, University of Pittsburgh, Pittsburgh, PA USA; ^2^ AdventHealth, Orlando, FL USA; ^3^ Faculty of Sport Sciences, Sport and Health University Research Institute (iMUDS), University of Granada., Granada, Granada Spain

## Abstract

Regular exercise has been shown to have beneficial effects including reducing risk of developing Alzheimer’s disease (AD). However, it is unclear if exercise has direct effects on AD pathophysiology. To this end, several emerging initiatives have been established to investigate plasma biomarker changes in relation to exercise among older adults. The aim of this talk is to described plasma biomarker results in two exercise‐based randomized controlled trials. The Investigating Gains in Neurocognition in an Intervention Trial of Exercise (IGNITE) is a completed multi‐site randomised dose‐response exercise trial of 12‐month duration that targeted over 600 cognitively normal 65–80‐year‐old adults in the United States. Participants were randomized into three different groups of exercise intensity. The second study is the Active Gains in brain Using Exercise During Aging (AGUEDA) is a 24‐week, single‐site two‐arm, single‐blinded trial that recrtuited n = 90 cognitively normal adults aged 65‐80 and living in Spain. In both studies, blood samples were collected at baseline as well as the end of the intervention. A mid‐study blood collection was further performed in IGNITE. Additionally, neuroimaging measures of amyloid and neurodegeneration were obtained, so were cognitive assessments using established tools. We performed measurements of plasma p‐tau217, p‐tau181, NfL, GFAP and amyloid beta 42/40 by immunoprecipiation‐mass spectrometry in all participants and at all timepoints. We will evaluate the time‐dependent profiles of these blood biomarkers, their associations with exercise intensity and neuroimaging markers, as well as potential moderating effects by demographic (e.g, sex, age, education, family history), and genetic factors (*APOE* e4 genotype). The results will be crucial to understanding associations between exercise and brain health.

